# Secondary Data Analyses of Subjective Outcome Evaluation Findings of the Project P.A.T.H.S. in Hong Kong

**DOI:** 10.1100/tsw.2010.177

**Published:** 2010-11-04

**Authors:** Daniel T. L. Shek, Rachel C.F. Sun

**Affiliations:** ^1^ Department of Applied Social Sciences, Hong Kong Polytechnic University, Hong Kong, China; ^2^ Public Policy Research Institute, The Hong Kong Polytechnic University, Hong Kong, China; ^3^ Kiang Wu Nursing College of Macau, Macau, China; ^4^ Division of Adolescent Medicine, Department of Pediatrics, University of Kentucky College of Medicine, Lexington, Kentucky, USA; ^5^ Division of Learning, Development and Diversity, Faculty of Education, University of Hong Kong, Hong Kong, China

**Keywords:** positive youth development, secondary data analyses, subjective outcome evaluation

## Abstract

The Project P.A.T.H.S. (Positive Adolescent Training through Holistic Social Programmes) is a positive youth development program in Hong Kong. After completion of the program, program implementers were required to draw five conclusions based on the subjective outcome evaluation findings collected from the program participants and implementers as reported in the evaluation report. Secondary analyses of the data collected from 48 schools that had joined the Secondary 3 program showed that most of the conclusions concerning perceptions of the program, instructors, and effectiveness of the program were positive in nature. There were also conclusions indicating strengths and possible improvement of the program. The present findings are consistent with the previous findings that suggest that the Project P.A.T.H.S. is well received by the stakeholders and the program is beneficial to the development of Chinese adolescents in Hong Kong.

## INTRODUCTION

With reference to the intensification of adolescent developmental problems in Hong Kong, there are very few systematic and multiyear positive youth development programs in Hong Kong[1]. The existing youth enhancement programs commonly deal with isolated problems and issues in adolescent development (i.e., deficits-oriented programs) and they are relatively short term in nature. To promote holistic development among adolescents in Hong Kong, The Hong Kong Jockey Club Charities Trust initiated and launched a project entitled "P.A.T.H.S. to Adulthood: A Jockey Club Youth Enhancement Scheme". The word "P.A.T.H.S." denotes **P**ositive **A**dolescent **T**raining through **H**olistic **S**ocial Programmes. The Trust invited academics of five universities in Hong Kong to form a research team, with The Hong Kong Polytechnic University as the lead institution, in order to develop a multiyear universal positive youth development program to promote holistic adolescent development in Hong Kong, with an earmarked grant of HK$400 million for the original phase and HK$350 million for the extension phase. The background of the project can be seen in Shek[2,3] and Shek and Sun[4].

There are two tiers of programs (Tier 1 and Tier 2) in this project. The Tier 1 Program is a universal positive youth development program where students in Secondary 1–3 normally participate in a 20-h program in the school year at each grade. A review of the literature shows that promotion of adolescent development in the following domains is important: promotion of bonding, cultivation of resilience, promotion of social competence, promotion of emotional competence, promotion of cognitive competence, promotion of behavioral competence, promotion of moral competence, cultivation of self-determination, promotion of spirituality, development of self-efficacy, development of a clear and positive identity, promotion of beliefs in the future, provision of recognition for positive behavior, provision of opportunities for prosocial involvement, and fostering prosocial norms[5]. To help adolescents develop in a holistic manner, these 15 adolescent developmental constructs are covered in the project, particularly in the Tier 1 Program. The conceptual model of the project can be seen in Shek[2].

Consistent with the spirit of postpositivism, a wide range of evaluation strategies were used to evaluate the effectiveness of the Tier 1 Program[4,6,7,8,9,10]. In particular, subjective outcome evaluations based on the program participants (Form A data) and program implementers (Form B data) were separately assessed. Actually, in the final evaluation report submitted to the research team, program implementers were required to integrate both sets of findings and write down five conclusions regarding the program's effectiveness. While there are arguments against the use of subjective outcome evaluation, there was substantial convergence between subjective outcome and objective outcome evaluation findings[11]. Furthermore, it can be argued that secondary data analyses based on the reports submitted by the program implementers can give a more comprehensive picture of the effectiveness of the program.

In the study conducted by Shek[12], the program implementers were invited to write down five conclusions based on an integration of the evaluation findings based on the subjective outcome evaluation findings collected from the program participants (Form A data) and program implementers (Form B data) for the Secondary 1 Program. Results showed that most of the conclusions concerning perceptions of the Tier 1 Program, instructors, and effectiveness of the programs were positive in nature. Although there were conclusions reflecting the respondents' appreciation of the program, responses on the difficulties encountered and suggestions for improvements were also observed. Similar findings were reported in Shek and Sun[13] based on the data collected from Secondary 1 students.

Although the above two studies clearly showed that the findings based on subjective outcome evaluation were quite positive, one limitation is that the findings were based on Secondary 1 students only. Although junior secondary school students are encountering several developmental challenges, such as adjustment to puberty, cognitive maturation, rapid expansion of social circle, increased stress, higher levels of social expectations, and gradual detachment from the family, there are comparatively more developmental challenges for Secondary 3 students as compared with their Secondary 1 and 2 counterparts in Hong Kong. Furthermore, previous studies showed that when students reach Secondary 3, they show poorer psychological well-being and adaptation skills, and they perceive family functioning to be poorer and parental control to be looser. As such, it is important to ask whether Secondary 3 students would have unique reactions to positive youth development programs and whether the program implementers would have particular feelings and comments about teaching Secondary 3 students.

The study described in this paper attempted to replicate the previous studies in examining the effectiveness of the Tier 1 Program of the Project P.A.T.H.S. in its third year of implementation in the Experimental Implementation Phase based on the secondary data analyses of conclusions drawn by implementers executing the Secondary 3 curriculum. As the students who joined the Project P.A.T.H.S. in January 2006 in the Secondary 1 level in the Experimental Implementation Phase entered their third year of the project in 2008, it is worthwhile to know the perceived program effects of the Project P.A.T.H.S. in Hong Kong. As pointed out by Fahs et al.[14], "replication of research is essential to the building and continued development of the scientific basis of any discipline" (p. 67). As such, the replication based on Secondary 3 students can give a picture on the generalizability of the findings across time.

There were several objectives of this study. First, it attempted to provide an integrated picture of the evaluation of the program based on the subjective outcome evaluation data collected from the program participants (Form A) and program implementers (Form B). Second, integrated views on the program implementers based on the program participants (Form A) and program implementers (Form B) were examined. Third, conclusions regarding the perceived effectiveness based on Forms A and B data were presented. Finally, integrated conclusions regarding recommendations on the program were explored.

## METHODS

In the 2007/08 school year, 48 schools joined the Secondary 3 level of the Project P.A.T.H.S. in the Experimental Implementation Phase. After the completion of the Tier 1 Program, students and implementers were invited to complete a subjective outcome evaluation questionnaire. A total of 6,830 students (with an average of 142.29 students per school, ranging from 23 to 213 students) and 286 implementers (teachers and social workers) responded to the Subjective Outcome Evaluation Form for Students (Form A) and Subjective Outcome Evaluation Form for Instructors (Form B), respectively.

Based on the evaluation data collected, the responsible worker in each school was required to complete an evaluation report where the quantitative and qualitative findings based on Forms A and B were summarized and described. In the last section of the report, the worker preparing the report was requested to write down the five most important conclusions they wished to draw regarding the evaluation of the program, which could give an overall picture regarding the perceived perceptions of the Tier 1 Program.

In brief, Form A assesses (a) participants' perceptions of the program, (b) participants' perceptions of the workers, (c) participants' perception of the effectiveness of the program, (d) participants willingness to recommend the program to other people with similar needs, (e) the extent to which the participants would join similar programs in the future, and (f) overall satisfaction with the program. There are also open-ended questions asking the participants' gains from the program, their appreciation of the program, their opinion about instructors, as well as areas for improvement. Similarly, Form B includes the evaluation of (a) program implementers' perceptions of the program, (b) program implementers' perceptions of their own practice, (c) implementers' perceptions of the effectiveness of the program, (d) the extent to which the implementers would recommend the program to other students with similar needs, (e) the extent to which the implementers would teach similar programs in the future, and (f) implementers' overall satisfaction with the program. Open-ended questions were asked regarding the things that the implementers learned in the program, things the implementers appreciated most, difficulties encountered, and areas that required improvement. Detailed design of Forms A and B can be referred to previous papers concerning the evaluation of the Project P.A.T.H.S.[13,15].

The data generated from the five conclusions were analyzed using general qualitative analyses techniques[16] by two research assistants, both having a Bachelor's Degree of Psychology. The final coding and categorization were further cross-checked by a colleague with a Master's Degree of Social Work. There were three steps in the data analysis process. First, raw codes were developed for words, phrases, and/or sentences that formed meaningful units in each conclusion at the raw responses level. Second, the codes were further combined to reflect higher-order attributes at the category of codes level. For example, the response of "students were satisfied with the instructors' performance" at the raw response level could be subsumed under the category of "satisfaction level", which could be further subsumed under the broad theme of "views on the program implementers" (see Table 2).

In order to minimize the possible biases involved, both intra- and inter-rater reliability on the coding were calculated. For intrarater reliability, each of the two research assistants who were primarily responsible for coding coded 20 randomly selected responses without looking at the original codes given after checking by the author. For inter-rater reliability, another two research assistants with Master's degrees who had not been involved in the data analyses coded the same 20 randomly selected responses independently without knowing the original codes given at the end of the scoring process after checking by the first author.

Following the principles of qualitative analyses proposed by Shek et al.[17], the following attributes of the study regarding data collection and analyses are highlighted. First, a general qualitative orientation is adopted. Second, the sources of data (e.g., number of participants) for analyses are described. Third, the issues of biases and ideological preoccupation are addressed. Fourth, inter- and intrarater reliability information is presented. Fifth, the categorized data is kept by a systematic filing system in order to ensure that the findings are auditable. Finally, possible explanations, including alternative explanations, are considered.

## RESULTS

Among the 48 participating schools, 240 conclusions were drawn from 48 evaluation reports and 516 meaningful units were extracted. These raw responses were further categorized into several categories, including views of the stakeholders on the program (Table 1), views of stakeholders on the program implementers (Table 2), perceived general and specific effectiveness of the program (Table 3), and recommendations toward the program (Table 4).

Regarding the conclusions related to the stakeholders' perceptions of the program, results in Table 1 show that most of the responses were positive in nature in the areas of satisfaction, program content, activity format, program arrangement, and program implementation. Among the 153 responses, 113 responses were classified as positive (73.86%). Examples of raw responses included "instructors regarded the program content to be comprehensive and diversified", "most students appreciated the objectives of the program", and "students regarded the atmosphere was good during program implementation". The intra- and inter-rater agreement percentages on the positivity of coding were both 100%.

For the perceptions of the program implementers, findings in Table 2 show that most of the responses were positive in nature. Among the 78 responses, 74 were positive (94.87%). The satisfaction level among stakeholders on the instructors' performance was very high. Over 60% (n = 47) of the responses were "students were satisfied with instructors' performance" or "instructors were satisfied with their own performance". The intra- and inter-rater agreement percentages on the positivity of the coding were also both 100%.

The findings on the perceived benefits of the program to the students are shown in Table 3. There was a total of 209 meaningful units that could be categorized in several levels, namely societal, familial, interpersonal, and personal. Overall, the positive effects of the program were evident; 194 out of 209 responses were positive (92.82%). A majority of responses (60.29%) regarding perceived program effectiveness were about students' personal growth; for instance, the program "enhanced students' development" (n = 49), "promoted students' abilities of differentiating between right and wrong" (n = 12), and "enhanced students' reflection of life" (n = 8). The intra- and inter-rater agreement percentages of the positivity of the coding were both 100%.

The suggestions for improvement can be seen in Table 4 (n = 76). It is noteworthy that some suggestions for improvement were contradictory (e.g., deepen program content vs. simplify and condense the program content). As it is difficult to determine whether the responses related to suggestions for improvement were really negative responses or not, positivity of the responses was not coded. Simply based on the category of code level, the intrarater agreement percentage was 100% and inter-rater agreement percentage was 85%.

## DISCUSSION

In the present study, findings on the conclusions drawn by the program implementers of the Tier 1 Program of the Project P.A.T.H.S. in the third year of the Experimental Implementation Phase via secondary data analyses are presented. Program implementers were asked to write down the five most important things they wanted to say about the program. About 40% of the responses (209 out of 516

**TABLE 1**
**Views of Stakeholders on the Program**

| Category | Responses | Nature of the Response | | | | Total |
|---|---|---|---|---|---|---|
| | | Positive | Neutral | Negative | Undecided | |
| Satisfaction Level | Positive impression toward the program | 10 | | | | 10 |
| | Satisfied with the program | 46 | | | | 46 |
| | Students were satisfied with their own performance in the program | 1 | | | | 1 |
| | Liked the program | 5 | | | | 5 |
| | Would join the program again | 1 | | | | 1 |
| | Would suggest friends to join the program | 1 | | | | 1 |
| | Neutral comments | | 4 | | | 4 |
| | Negative comments | | | 6 | | 6 |
| | **Subtotal** | **64** | **4** | **6** | **0** | **74** |
| Program Content | Comprehensive and systematic content | 1 | | | | 1 |
| | Impressive content | 3 | | | | 3 |
| | Liked the program design/objective | 7 | | | | 7 |
| | Clear objectives and strong theoretical support | 11 | | | | 11 |
| | Up-to-date and detailed information | 1 | | | | 1 |
| | Other positive comments | 7 | | | | 7 |
| | Neutral comments | | 10 | | | 10 |
| | Too broad theoretical support | | | 1 | | 1 |
| | Too much/poor content | | | 4 | | 4 |
| | Overlapping | | | 2 | | 2 |
| | Lacked English curriculum | | | 1 | | 1 |
| | Unable to match students' interest and abilities | | | 1 | | 1 |
| | **Subtotal** | **30** | **10** | **9** | **0** | **49** |
| Activity Format | Diversified teaching means | 1 | | | | 1 |
| | Interesting/sufficient teaching materials/interactive activities | 5 | | | | 5 |
| | Liked the games | 1 | | | | 1 |
| | Too many tasks or written tasks | | | 2 | | 2 |
| | **Subtotal** | **7** | **0** | **2** | **0** | **9** |
| Program Arrangement | Whole school cooperation | 1 | | | | 1 |
| | Flexible program arrangement | 1 | | | | 1 |
| | **Subtotal** | **2** | **0** | **0** | **0** | **2** |
| Program Implementation | Good atmosphere | 4 | | | | 4 |
| | Real cases sharing | 2 | | | | 2 |
| | Sufficient discussion time | 1 | | | | 1 |
| | Interactive | 2 | | | | 2 |
| | Other positive comment | 1 | | | | 1 |
| | Neutral comments | | 5 | | | 5 |
| | Time constraint | | | 4 | | 4 |
| | **Subtotal** | **10** | **5** | **4** | **0** | **19** |
| **Total Responses** | | **113** | **19** | **21** | **0** | **153** |

**TABLE 2**
**Views of Stakeholders on Program Implementers**

| Category | Responses | Nature of the Response | | | | Total |
|---|---|---|---|---|---|---|
| | | Positive | Neutral | Negative | Undecided | |
| Satisfaction Level | Students were satisfied with instructors' performance | 22 | | | | 22 |
| | Instructors were satisfied with their own performance | 25 | | | | 25 |
| | Positive comments | 5 | | | | 5 |
| | **Subtotal** | **52** | **0** | **0** | **0** | **52** |
| Views about the Instructors | Professional attitude | 1 | | | | 1 |
| | Commitment | 4 | | | | 4 |
| | Instructor's attitude and performance influenced students' learning | 7 | | | | 7 |
| | Cared about students | 3 | | | | 3 |
| | Helped students | 3 | | | | 3 |
| | Sufficient teaching skills | 1 | | | | 1 |
| | **Subtotal** | **19** | **0** | **0** | **0** | **19** |
| Others | Other positive comments | 3 | | | | 3 |
| | Neutral comments | | 4 | | | 4 |
| | **Subtotal** | **3** | **4** | **0** | **0** | **7** |
| **Total Responses** | | **74** | **4** | **0** | **0** | **78** |

meaningful units) were related to the program effectiveness, 30% (153 out of 516) were about views toward the program, and 15% concerned implementers' performance. These findings echo the previous evaluation studies of the Project P.A.T.H.S. using the same methodology[12,13], as well as using other evaluation methods[9,22,25] that suggest that the Tier 1 Program of the Project P.A.T.H.S. is perceived as beneficial to the development of the program participants.

Although the present findings can be interpreted as evidence supporting the effectiveness of the Project P.A.T.H.S., several alternative explanations exist. The first alternative explanation is that the findings are due to insufficient evaluation expertise of the program implementers. However, this assumption can be eliminated because implementers (social workers and teachers) learn about program evaluation in their professional training. For those who joined the Project P.A.T.H.S., training on data collection and report writing was provided in the training program. In addition, according to the utilization-focused approach of evaluation[18], views of the program implementers and the reports should be regarded as having a strong weight because they are professional inside the implementation process and are knowledgeable about the program. Furthermore, evaluation manuals with report templates were provided to all participating schools, which helped program implementers to familiarize themselves with the steps of data collection, analyses, and report writing. In short, it can be argued that the program implementers were able to integrate the subjective outcome evaluation findings and translate them into meaningful conclusions, and thus credibility of the data collection and reports is high.

The second alternative explanation is that the findings were due to biases, such as cognitive dissonance, rice bowl, and revenge arguments[11,15]. However, an examination of the present findings showed that the conclusions drawn by the program implementers were in line with other evaluation findings reported by the research team. Since an integration of different existing findings has painted a consistent picture of program effectiveness based on the principle of triangulation, it indicates that the influences of biases are minimal.

**TABLE 3**
**Perceived Effectiveness of the Program**

| Category | Subcategory | Responses | Nature of the Response | | | | Total |
|---|---|---|---|---|---|---|---|
| | | | Positive | Neutral | Negative | Undecided | |
| Societal Level | Social Responsibility | Enhanced students' social participation and sense of caring | 11 | | | | 11 |
| | | **Subtotal** | **11** | **0** | **0** | **0** | **11** |
| Familial Level | Family Relationships | Enhanced the relationship between students and their families | 1 | | | | 1 |
| | | **Subtotal** | **1** | **0** | **0** | **0** | **1** |
| Interpersonal Level | General Interpersonal Competence | Improved interpersonal relationship | 1 | | | | 1 |
| | | Enhanced bonding with healthy adults and schoolmates | 1 | | | | 1 |
| | | Enhanced instructors and students relationship | 2 | | | | 2 |
| | | Enhanced peer relationship | 2 | | | | 2 |
| | | Increased communication between students | 1 | | | | 1 |
| | | Increased communication between instructors and students | 1 | | | | 1 |
| | | **Subtotal** | **8** | **0** | **0** | **0** | **8** |
| | Specific Interpersonal Competence | Promoted communication skills | 2 | | | | 2 |
| | | Enhanced understanding/ mutual understanding | 6 | | | | 6 |
| | | Enhanced ability of cooperating with others | 1 | | | | 1 |
| | | Enhanced social skills | 13 | | | | 13 |
| | | **Subtotal** | **22** | **0** | **0** | **0** | **22** |
| Personal Level | Cherishing Life | Treasuring of life | 3 | | | | 3 |
| | | Reflection of life | 8 | | | | 8 |
| | | **Subtotal** | **11** | **0** | **0** | **0** | **11** |
| | Cognitive Competence | Promoted analytical ability | 4 | | | | 4 |
| | | Enhanced self-reflection | 3 | | | | 3 |
| | | **Subtotal** | **7** | **0** | **0** | **0** | **7** |

*Table 3 continues*

**TABLE 3 (continued)**

| Category | Subcategory | Responses | Nature of the Response | | | | Total |
|---|---|---|---|---|---|---|---|
| | | | Positive | Neutral | Negative | Undecided | |
| | Positive Self-Image | Enhanced students' development | 49 | | | | 49 |
| | | Cultivation of resilience | 4 | | | | 4 |
| | | Mastering of future | 7 | | | | 7 |
| | | Enhanced self-confidence | 5 | | | | 5 |
| | | Enhanced self-determination | 5 | | | | 5 |
| | | Enhanced self-understanding | 7 | | | | 7 |
| | | **Subtotal** | **77** | **0** | **0** | **0** | **77** |
| | Emotional Competence | Promoted emotional control | 8 | | | | 8 |
| | | **Subtotal** | **8** | **0** | **0** | **0** | **8** |
| | Goal Setting | Goal setting | 2 | | | | 2 |
| | | Future planning | 4 | | | | 4 |
| | | **Subtotal** | **6** | **0** | **0** | **0** | **6** |
| | Moral Competence and Virtues | Promoted ability of differentiating between right and wrong | 12 | | | | 12 |
| | | Strengthened positive values | 1 | | | | 1 |
| | | **Subtotal** | **13** | **0** | **0** | **0** | **13** |
| | Learning | Students gained extracurricular knowledge | 1 | | | | 1 |
| | | Enhanced students' participation in classroom | 3 | | | | 3 |
| | | **Subtotal** | **4** | **0** | **0** | **0** | **4** |
| Others | | Could help instructors | 4 | | | | 4 |
| | | Other positive impacts | 22 | | | | 22 |
| | | Other negative comments | | | 15 | | 15 |
| | | **Subtotal** | **26** | **0** | **15** | **0** | **41** |
| **Total Responses** | | | **194** | **0** | **15** | **0** | **209** |

There are several advantages of using qualitative secondary data analysis as demonstrated in this study. First of all, it saves time, money, and effort of data collection. Also, the database is large and readily accessible. Moreover, the information regarding the data collection process is available and documented. In addition, the data collected by open-ended questions are not limited by the preset question-and-answer format of the survey data, and are not affected by the possible investigator's biases appearing in the interviews. Most important of all, it engages the program implementers as evaluators who are important stakeholders of the project.

However, several criticisms could be leveled against studies that utilize secondary data analyses such as the present study. First, since secondary data analyses utilize an existing database, it is not possible to have interactive collaboration with the program implementers to explore further related issues. Second,

**TABLE 4**
**Recommendations toward the Program**

| Category | Description | Total |
|---|---|---|
| Program Content | Add interesting elements | 2 |
| | Content should be adjusted to suit the needs/interests/abilities of students | 8 |
| | Deepen program content | 7 |
| | Simplify and condense the program content | 3 |
| | Be more applicable to real-life situations | 4 |
| | Match up with the social environment | 3 |
| | Need diversified content | 4 |
| | Content should be more lively | 1 |
| | Other comments related to content | 3 |
| | **Subtotal** | **35** |
| Program Format | Add more games/activities | 9 |
| | Add more multimedia | 4 |
| | Need more diversified format | 3 |
| | Improve growth puzzle | 1 |
| | Enhance students' self-reflection and sharing | 1 |
| | Set up reward and penalty systems | 1 |
| | Other comments related to the program format | 4 |
| | **Subtotal** | **23** |
| Time Arrangement | Match up content and time | 5 |
| | Prolong duration of lesson | 3 |
| | Reduce duration of lesson | 2 |
| | **Subtotal** | **10** |
| Implementation | Strengthen follow-up and consolidation work | 5 |
| | **Subtotal** | **5** |
| Others | Other recommendations | 3 |
| | **Subtotal** | **3** |
| **Total Responses** | | **76** |

the conclusions written were brief, and could hardly provide an in-depth understanding of the implementation process or school administration. Therefore, it is valuable to conduct school-based case studies to document exemplary school administration and program implementation. Third, although the five conclusions generated from each evaluation report gave an overall picture of each school, they could not give detailed descriptions of the perceptions of individual program implementers and participants. Thus, it is more illuminating to carry out in-depth interviews with instructors and students. Despite these limitations, the existing research findings suggest that the Tier 1 Program is well received by both program participants and implementers, and it is regarded as helpful to the students' overall development. These findings are consistent with those reported in the literature[19,20,21,22,23,24,25,26].

## ACKNOWLEDGMENTS

The authorship of this paper is equally shared by both authors. The Project P.A.T.H.S. and preparation for this paper was financially supported by The Hong Kong Jockey Club Charities Trust.
